# Spatiotemporal Dynamics of SARS-CoV-2 Variants During the First Year of the Pandemic Highlight the Earlier Emergence of the Zeta Variant of Interest in Brazil

**DOI:** 10.3390/pathogens13121069

**Published:** 2024-12-06

**Authors:** Marília Mazzi Moraes, Guilherme Rodrigues Fernandes Campos, Cecília Artico Banho, Alice Freitas Versiani, Thayza Maria Izabel Lopes dos Santos, Maisa Carla Pereira Parra, Edoardo Lobl, Tayna Manfrin Galvão, Nikos Vasilakis, Maurício Lacerda Nogueira

**Affiliations:** 1 Laboratório de Pesquisas em Virologia, Departamento de Doenças Infecciosas e Parasitárias, Faculdade de Medicina de São José do Rio Preto (FAMERP), São José do Rio Preto 15090-000, SP, Brazil; mariliamazzi@hotmail.com (M.M.M.); guilhermecampos07@gmail.com (G.R.F.C.); ceci.abanho@gmail.com (C.A.B.); thayza@gmail.com (T.M.I.L.d.S.); maisapparra@hotmail.com (M.C.P.P.); edoardo.lobl@gmail.com (E.L.); tayy-g@hotmail.com (T.M.G.); 2 Department of Pathology, University of Texas Medical Branch (UTMB), Galveston, TX 7555-0609, USA; afversia@utmb.edu (A.F.V.); nivasila@utmb.edu (N.V.)

**Keywords:** SARS-CoV-2, variant of interest, genomic surveillance

## Abstract

During the COVID-19 pandemic, SARS-CoV-2 caused an alarming number of cases and deaths worldwide. Brazil was severely affected from late 2020 onward, especially after the emergence of variants of concern (VOCs) and variants of interest (VOIs). Although much is known about the dynamics and evolution of SARS-CoV-2 VOIs and VOCs in the country, information is still lacking on how the cocirculation of several SARS-CoV-2 lineages, along with the lack of vaccination and low adherence to social isolation measures, shaped the first year of the COVID-19 pandemic in Brazil. We used a combination of genomic and epidemiological data to understand the transmission dynamics of SARS-CoV-2 variants from March to November 2020 within a medium-sized city in São Paulo state. By generating 627 SARS-CoV-2 whole genomes, we were able to identify 10 different SARS-CoV-2 lineages that were cocirculating in the municipality. Although many introduction events have been identified, B.1.1.28 and B.1.1.33 variants were the most frequent during the sampling period. We also detected the presence of the Zeta and N.9 variants earlier than had previously been reported in Brazil. These findings reinforce the need for active genomic surveillance to detect new viral introductions that may impact health systems during public health emergencies.

## 1. Introduction

SARS-CoV-2 was detected in Wuhan, China, in December 2019 and rapidly spread to other countries a few months later [1,2]. The virus was responsible for more than 776 million disease cases and 7 million deaths worldwide by September 2024. Even though the end of the public health emergency of international concern was declared in May 2023, COVID-19 still remains an ongoing health problem that needs to be constantly monitored. Several SARS-CoV-2 variants emerged during the course of the COVID-19 pandemic; these lineages were classified based on the constellation of mutations they harbored. Those presenting increases in transmissibility, pathogenesis, and immune evasion were named variants of interest (VOIs), such as Epsilon, Zeta, Eta, Theta, Iota, Kappa, Lambda, and Mu, and variants of concern (VOCs), such as Alpha, Beta, Gamma, Delta, and Omicron [3]. The emergence of VOIs and VOCs attracted attention due to their role in increasing the number of COVID-19 cases, leading to subsequent waves of infection and healthcare system overloads around the world.

Brazil was severely affected by the COVID-19 pandemic and was an epicenter of disease cases and deaths as early as March 2020. In July 2020, the country was classified as second in the number of COVID-19 cases worldwide [4]. SARS-CoV-2 disseminated over the entirety of Brazil after being introduced in the city of São Paulo in February 2020 [5]. During the first year of the pandemic, multiple SARS-CoV-2 lineages cocirculated [6]; however, this scenario changed with the emergence of VOIs and VOCs in late 2020 [7,8]. Most of the COVID-19 cases and deaths occurred in the Southeast region, which includes the states of São Paulo (SP), Rio de Janeiro (RJ), Minas Gerais (MG), and Espírito Santo (ES) [9]. São José do Rio Preto was an important city within the context of the pandemic, with the third-highest number of COVID-19 cases and deaths in São Paulo state [10].

Although much is known about the course of the COVID-19 pandemic from the emergence of VOCs onward, very little information is available about the spread and evolution of SARS-CoV-2 in early 2020 in Brazil. Additionally, there is a lack of genome sequence data from this period, which limits the understanding of how the cocirculation of several variants, combined with the absence of vaccination, shaped the progression of the pandemic in the country. Studies associating sequencing and epidemiological data are essential to address this gap. Here, we investigated the spatiotemporal dynamics of SARS-CoV-2 variants from March to November 2020 in a medium-sized city that can be considered to reflect the Brazilian epidemiological landscape.

## 2. Materials and Methods

### 2.1. Study Area

São José do Rio Preto (SJdRP) is situated in the Northeast region of São Paulo state, Brazil (20°49′11″ S and 49°22′46″ W) and covers a total area of 431,944 km^2^. In 2022, the city’s population was estimated at 480,393 inhabitants. The municipality harbors the Hospital de Base de São José do Rio Preto (HB), a reference health center in the state that serves more than two million inhabitants and was considered the second-largest intensive care unit for COVID-19 in Brazil during the pandemic [11]. Additionally, HB is an academic medical center of Faculdade de Medicina de São José do Rio Preto (FAMERP), where Laboratório de Pesquisas em Virologia (LPV) is located.

### 2.2. Ethical Aspects and Sampling

The present study was conducted at the LPV and approved by the FAMERP Ethics Review Board (31588920.0.0000.5415, approved on 14 May 2020). Because all data were analyzed anonymously, informed consent from the participants was not required. Convenience samples of SJdRP residents showing positive molecular diagnosis for COVID-19 were randomly selected for whole-genome sequencing. A total of 627 nasopharyngeal swab samples collected between March and November 2020 were selected based on the cycle threshold value (≤34) and availability of epidemiological metadata, such as date of sample collection and municipality of residence.

### 2.3. Molecular Investigation and Whole-Genome Sequencing

Viral RNA was extracted using Quick-RNA™ Viral Kit (Zymo Research, Irvine, CA, USA), according to the manufacturer’s instructions. The presence of SARS-CoV-2 RNA was investigated via real-time reverse transcription polymerase chain reaction (RT-qPCR) using Allplex™ 2019-nCoV Assay (Seegene, Contagem, MG, Brazil). Primers and probes designed by the manufacturer targeted the viral envelope protein (E), nucleocapsid protein (N), and RNA-dependent RNA polymerase (RdRp). The RT-qPCR was conducted in QuantStudio 5 Real-Time PCR System (ThermoFisher Scientific, Waltham, MA, USA), and the results were analyzed in QuantStudio 5 software v1.5.1 (Thermo Fisher Scientific, MA, USA), with a cycle threshold value (Ct) of ≤40 considered positive.

Whole-genome amplification and library preparation were performed with Illumina CovidSeq Test (Illumina Inc., San Diego, CA, USA) and QIAseq SARS-CoV-2 Primer Panel (Qiagen, Germantown, MD, USA), according to the manufacturer’s instructions. Library quality and size were verified using Agilent 4150 TapeStation (Agilent Technologies Inc., Santa Clara, CA, USA). Library quantification was performed in Qubit 3.0 fluorimeter (ThermoFisher Scientific, MA, USA) using Qubit dsDNA HS assay kit (ThermoFisher Scientific, MA, USA). Sequencing was implemented in Illumina MiSeq System (Illumina Inc., USA), using 300-cycle MiSeq Reagent Kit v3 (2 × 150 bp cycles) (Illumina Inc., USA). 

### 2.4. Genome Assembly and Lineage Assignment

FASTQ files were processed with the ARTIC Nextflow pipeline (https://github.com/connor-lab/ncov2019-artic-nf/tree/illumina, accessed on 24 February 2022); only genomes presenting ≥90% coverage were selected for further analyses. The Pangolin web application v.1.21 (https://pangolin.cog-uk.io/, accessed on 24 February 2022) was used for lineage classification [12].

### 2.5. Geoprocessing

A database of SARS-CoV-2 identified lineages, geographic coordinates, and sample collection dates was created. The maps were generated using QGIS v.3.36.0-1 software (http://qgis.org, accessed on 4 March 2023). The shapefiles used in this study are available from the Brazilian Institute for Geography and Statistics (IBGE) at https://www.ibge.gov.br/geociencias/organizacao-do-territorio/malhas-territoriais/15774-malhas.html, accessed on 4 March 2023.

### 2.6. Phylogenetic and Phylogeographic Analyses

The datasets used for the analyses (DOI: 10.17632/wjyjh9vvmw.4) comprise the complete SARS-CoV-2 genomes generated in this study, as well as complete SARS-CoV-2 genomes retrieved from the GISAID database (https://gisaid.org, accessed on 10 October 2023) [Supplementary Tables S1–S6] [13]. Nucleotide sequences were aligned using MAFFT v.7.520 (https://mafft.cbrc.jp/alignment/software/, accessed on 10 October 2023) [14]. Maximum Likelihood (ML) trees were reconstructed in IQ-TREE v.2.2.0 (http://www.iqtree.org/, accessed on 1 November 2023). The best-fit model was selected by ModelFinder application according to the Bayesian Information Criterion (BIC) [15]. Branch support values were accessed using a combination of Ultrafast Bootstrap (UFBoot) and SH-like approximate likelihood ratio (SHaLRT) tests, each conducted with 10,000 replicates [16,17]. TreeTime v.0.9.3 was used to transform the raw ML trees into dated trees considering the SARS-CoV-2 evolutionary rate of 8.0 × 10^−4^ nucleotide substitutions per site per year [18]. The temporal signal of ML trees was evaluated using TempEst tool v.1.5.3 [19], considering a correlation coefficient >0.4 to accept the temporal structure. The final time-scaled tree topology for the B.1.1.28, B.1.1.33, and Zeta lineages was used to infer the number of viral exchange events that occurred in 2020 in the five Brazilian regions and SJdRP, using TreeTime mugration v.0.9.3.

The Bayesian analyses were performed using the Markov chain Monte Carlo (MCMC) method implemented in BEAST v1.10.4 (https://beast.community/, accessed on 1 November 2023), as described by Giovanetti et al. (2022) [6]. Briefly, selected parameters included GTR+I+G nucleotide substitution model, strict molecular clock, and Bayesian skyline model as the coalescent tree prior. Additionally, a flexible relaxed random walk diffusion model with Cauchy distribution and jitter window size of 0.01 was used to model the phylogenetic dispersion of the B.1.1.28, B.1.1.33, and Zeta lineages. We randomly selected sequences from different Brazilian locations of the three variants. The analyses were run for 250 million MCMC steps with sampling parameters every 25,000 generations. Results were visualized using Tracer v1.7.1 [20]. The Maximum Clade Credibility (MCC) trees were summarized using LogCombiner v1.10.4 and TreeAnnotator v1.10.4 after discarding the first 10% as burn-in. The spatiotemporal information in the posterior trees was extracted using SERAPHIM, an R software v4.2.3 (https://posit.co/download/rstudio-desktop/, accessed on 13 January 2024) package [21].

### 2.7. Mutation Analysis

To assess the presence of defining mutations in the SARS-CoV-2 genomes generated in this study, we used MALVIRUS v1.0.285 [22]. We assembled sequence datasets for the Zeta and N.9 lineages (DOI: 10.17632/wjyjh9vvmw.2). The datasets were compared against the SARS-CoV-2 reference genome (EPI_ISL_402124), and the frequency occurrence of each mutation was identified for each lineage. The untranslated regions 5’UTR and 3’UTR of the SARS-CoV-2 genomes were not considered in this analysis once they were not obtained via sequencing.

## 3. Results

### 3.1. Sample Characterization

From February to December 2020, there were 36,185 confirmed COVID-19 cases and 1386 confirmed deaths in SJdRP [9] [Supplementary Figure S1]. The pandemic containment measures, such as the use of masks, closure of schools, and prohibition of agglomerations, started being implemented in the city in March 2020. Average social isolation rates began to decline in June, and in August, the city registered its highest numbers of COVID-19 cases and deaths [23] [Supplementary Figure S2]. Considering that SJdRP harbors HB, a reference center for COVID-19 care and treatment for more than 102 cities in the XV Regional Health District (RHD), spatiotemporal studies in the municipality are important to understand the transmission dynamics of SARS-CoV-2 during the first year of the pandemic, a period for which very little information is available.

We generated 627 SARS-CoV-2 whole genomes [Supplementary Table S9], which represent 91.67% of all the SARS-CoV-2 genomes of SJdRP sequenced in 2020 and available in the GISAID (https://gisaid.org/, accessed on 2 December 2024) database until October 2024. The collection dates ranged from March to November 2020. The samples were obtained from 293 males (n = 46.73%) and 334 females (n = 53.27%), all residents of SJdRP. Patient ages ranged from 1 to 94 years of age; however, the most sampled population was classified in the 40–44 age group [Supplementary Figure S3]. 

### 3.2. Lineage Assignment and Geoprocessing

**Figure 1:**
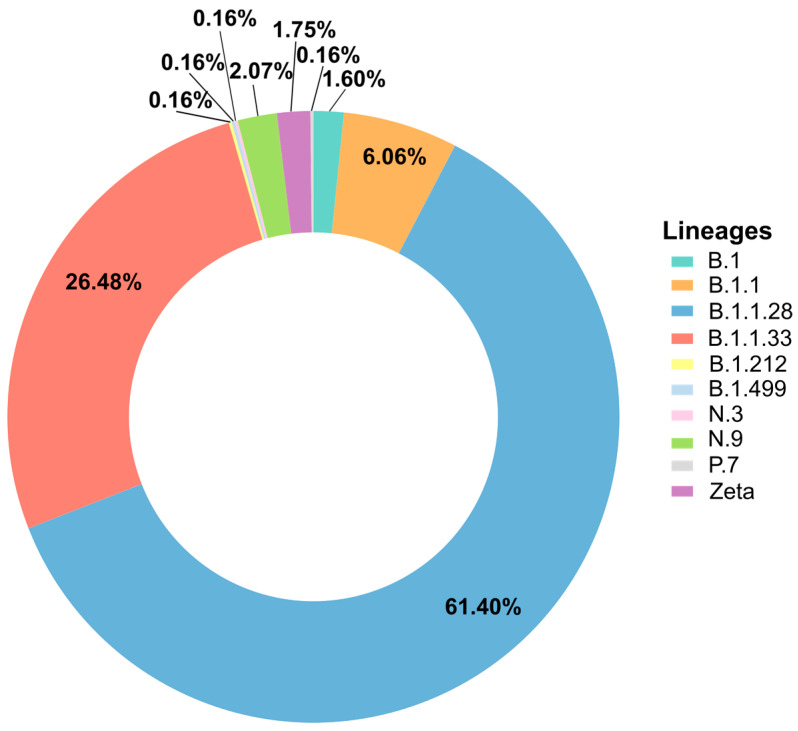
Lineages detected in SJdRP from March to November 2020. The figure shows the proportion of each lineage considering the 627 genomes obtained in this study.

According to the Pangolin v1.21 classification, the most prevalent SARS-CoV-2 lineage detected in SJdRP was B.1.1.28 (n = 61.40%), followed by B.1.1.33 (n = 26.48%), B.1.1 (n = 6.06%), N.9 (n = 2.07%), Zeta (n = 1.75%), B.1 (n = 1.59%), B.1.212 (n = 0.16%), B.1.499 (n = 0.16%), N.3 (n = 0.16%), and P.7 (n = 0.16%) [Figure 1].

**Figure 2:**
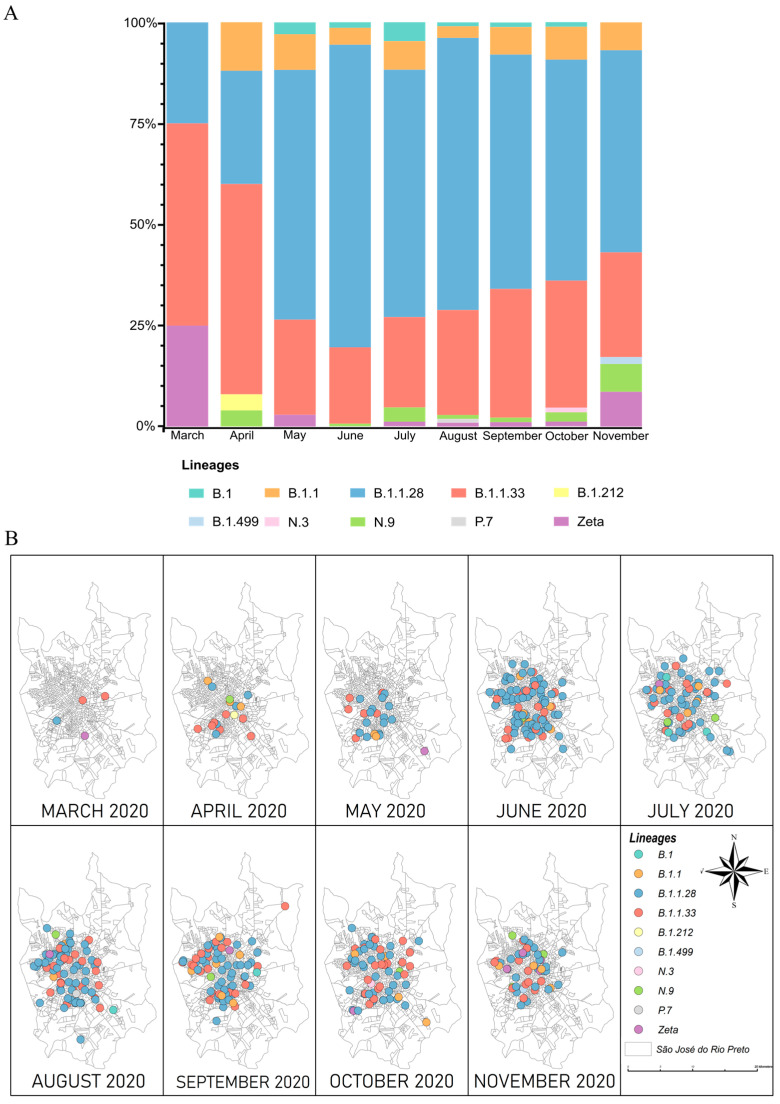
Geographic and temporal distribution of SARS-CoV-2 variants detected in SJdRP via genomic surveillance, March–November 2020. (**A**) Temporal distribution of SARS-CoV-2 lineages detected in SJdRP. (**B**) Geographic distribution of lineages in SJdRP by month of detection. Sampling location and collection dates were used to create the maps in QGIS v.3.36.0-1 software (http://qgis.org, accessed on 4 March 2023).

Data on the number of lineages identified monthly show that the B.1, B.1.1, B.1.1.28, B.1.1.33, N.9, and Zeta variants were detected throughout nearly all of the study period, while the B.1.212, B.1.499, N.3, and P.7 variants were detected only once [Supplementary Figures S4 and S5]. We noted that the B.1.1.33 lineage was the most frequent in March and April 2020, but B.1.1.28 became the dominant circulating SARS-CoV-2 lineage in the city from May 2020 [Figure 2A]. Moreover, a geoprocessing analysis showed that all the identified variants were widely distributed in the municipality [Figure 2B]. Interestingly, Zeta and N.9 were first detected in the city in March and April 2020, respectively.

### 3.3. Phylogenetic Analysis and Root-to-Tip Regression Analysis

We performed a phylogenetic analysis to understand the evolutionary history of SARS-CoV-2 in SJdRP. A time-scaled Maximum Likelihood (ML) tree was constructed using 620 complete genomes generated in this study, 38 SARS-CoV-2 whole genomes from residents of SJdRP retrieved from the GISAID database (https://gisaid.org/, accessed on 1 November 2023), the first SARS-CoV-2 genome detected in Brazil (EPI_ISL_413016), and the SARS-CoV-2 reference genome (EPI_ISL_402124). Therefore, the complete dataset includes sequences from March to December 2020 [Supplementary Table S1]. 

**Figure 3:**
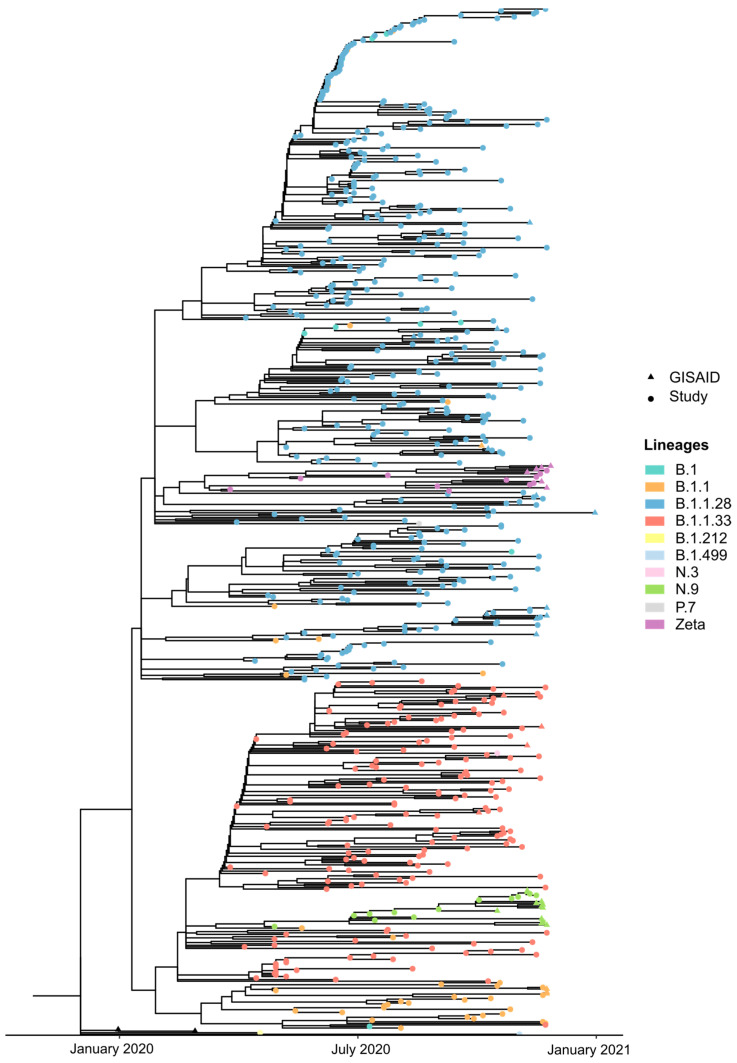
SARS-CoV-2 Maximum Likelihood (ML) analysis based on complete genome sequences from SJdRP. The phylogenetic tree was reconstructed using GTR+F+I+R4 as the best nucleotide substitution model. The reliability of branching patterns was tested by combining Ultrafast Bootstrap (UFBoot) and SH-like Approximate Likelihood-ratio (SH-aLRT) tests, with 10,000 replicates each. The analysis included a total of 658 genomes and was conducted in IQ-TREE v2.2.0. Tips are colored according to the SARS-CoV-2 Pangolin v1.21 lineage classification, and tip shapes represent genome source. The first SARS-CoV-2 genome detected in Brazil and the SARS-CoV-2 reference genome are shown as black triangles.

Our ML analysis showed that the Zeta and P.7 lineages clustered together with B.1.1.28, their ancestral lineage. Similarly, N.3 and N.9 grouped within the B.1.1.33 clade [Figure 3]. Lineages B.1 and B.1.1, which are ancestral to the main lineages that were circulating in 2020, were observed grouped in different clades. Furthermore, the B.1.212 and B.1.499 lineages were detected once, suggesting a single importation event.

**Figure 4:**
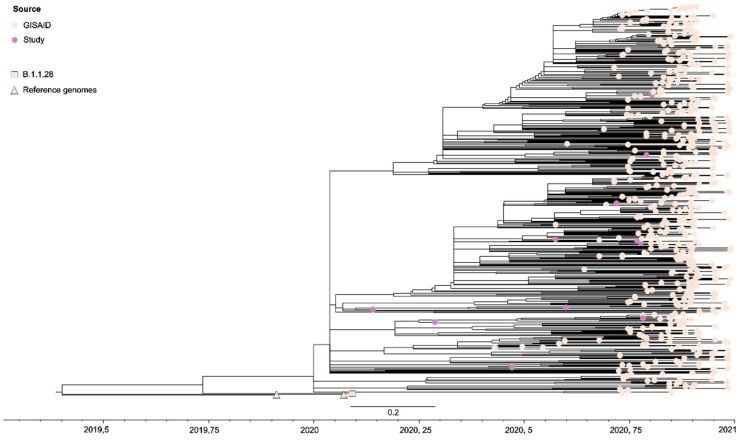
SARS-CoV-2 Maximum Likelihood (ML) analysis based on complete genome sequences of VOI Zeta. The phylogenetic analysis was conducted in IQ-TREE v2.2.0. The phylogenetic tree was reconstructed using GTR+F+I+G4 as the best nucleotide substitution model. The reliability of branching patterns was tested by combining Ultrafast Bootstrap (UFBoot) and SH-like Approximate Likelihood-ratio (SH-aLRT) tests, with 10,000 replicates each. The dataset used comprises a total of 583 genomes, and tips are colored according to genome source. The first B.1.1.28 genome detected in Brazil is shown as a square and the SARS-CoV-2 reference genomes are shown as triangles.

An interesting finding was the early detection of VOI Zeta (March 2020) in SJdRP. Because of its status as a variant of interest and its impact on the number of COVID-19 cases, we opted to specifically investigate the circulation dynamics of the Zeta variant in the city [Supplementary Figure S6]. We built a Maximum Likelihood (ML) tree to estimate the emergence of VOI Zeta using 11 complete genomes generated in this study and 572 complete genomes retrieved from the GISAID database (https://gisaid.org/, accessed on 1 November 2023), including the first SARS-CoV-2 genome detected in Brazil (EPI_ISL_413016), the first SARS-CoV-2 B.1.1.28 lineage genome detected in the country (EPI_ISL_416036), and the SARS-CoV-2 reference genome (EPI_ISL_402124) [Figure 4]. The dataset comprises sequences from SJdRP and all Brazilian regions from March 2020 to January 2021 [Supplementary Table S2]. 

Our analysis revealed that the Zeta sequences generated in the present study were interspersed with sequences from Rio de Janeiro, as well as those from various geographic regions in Brazil. This finding suggests multiple introduction events in SJdRP [Supplementary Figure S7]. Another noteworthy observation is that the Zeta genomes obtained in this study exhibited genetic divergence, as indicated by branch length in the phylogeny and the root-to-tip genetic distance analysis [Supplementary Figures S7 and S8], similar to genome sequences collected in the latter half of 2020. This implies that the Zeta variant was circulating at a lower frequency earlier than previously reported. However, due to the limited SARS-CoV-2 sequencing efforts in early 2020, its presence may not have been detected. To further investigate the origin of the Zeta variant, we mapped its location to both the tips and internal nodes of the tree topology. We observed that, although the Zeta variant was first detected in SJdRP in March 2020, its emergence likely took place in Rio de Janeiro state. It is important to highlight that a limitation of the present study is the small number of VOI Zeta sequences at the beginning of 2020 from different geographic locations, which made it difficult to estimate the variant divergence time robustly.

We also performed a phylogenetic analysis of the N.9 variant. We built a Maximum Likelihood (ML) tree using 13 complete genomes generated in this study and 166 complete genomes retrieved from the GISAID database (https://gisaid.org/, accessed on 7 October 2024), including the first SARS-CoV-2 genome detected in Brazil (EPI_ISL_413016), the first SARS-CoV-2 B.1.1.33 lineage genome detected in the country (EPI_ISL_476221), and the SARS-CoV-2 reference genome (EPI_ISL_402124) [Supplementary Figure S9]. The dataset comprises sequences from SJdRP and all Brazilian regions from April 2020 to December 2020 [Supplementary Table S6]. 

As observed for VOI Zeta, the N.9 variant sequences detected in SJdRP was interspersed with sequences from different geographic regions of Brazil. The N.9 variant genomes obtained in this study also exhibited genetic divergence, as indicated by branch length in the phylogeny and the root-to-tip genetic distance analysis [Supplementary Figures S9 and S10], similar to genome sequences collected in the latter half of 2020. Our results indicate that, as the Zeta variant, N.9 was circulating at a lower frequency earlier than previously reported in Brazil.

### 3.4. Spatiotemporal Dispersion of the B.1.1.28, B.1.1.33, and Zeta Lineages

**Figure 5:**
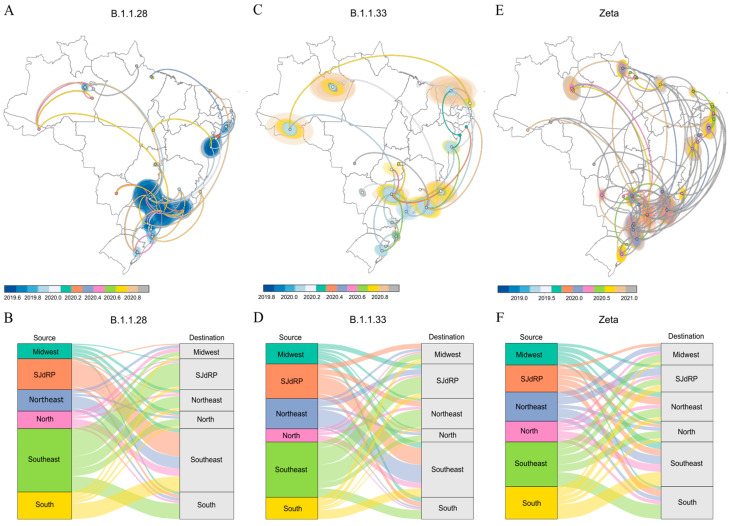
Spatiotemporal spread of the B.1.1.28, B.1.1.33, and Zeta lineages in Brazil. Phylogeographic reconstruction of the spread of (**A**) B.1.1.28, (**C**) B.1.1.33 and (**E**) Zeta across Brazil. Circles represent nodes of the maximum clade credibility phylogeny and are colored according to their inferred time of occurrence. Shaded areas represent the 80% highest posterior density interval and depict the uncertainty of the phylogeographic estimates for each node. Solid curved lines indicate links between nodes and direction of movement. Number of viral exchanges of (**B**) B.1.128, (**D**) B.1.1.33 and (**F**) Zeta, between SJdRP and the five Brazilian regions (North, Northeast, Midwest, Southeast, and South).

To better understand the transmission dynamics during the first year of the COVID-19 pandemic, we investigated the spatiotemporal dispersion of the most prevalent lineages detected in SJdRP, B.1.1.28 and B.1.1.33, as well as VOI Zeta, due to evidence of its early emergence in 2020 [Figure 5A,C,E]. Each dataset comprised genomes generated in this study and genomes retrieved from the GISAID database (https://gisaid.org/, accessed on 1 November 2023) from all Brazilian capitals [Supplementary Tables S3–S5]. Our results revealed that SJdRP contributed to the spread of variants B.1.1.28, B.1.1.33, and Zeta throughout Brazil, since viral importation and exportation events were observed between SJdRP and all five Brazilian regions [Figure 5B,D,F].

B.1.1.28 was the most prevalent lineage detected in SJdRP during 2020. Our spatiotemporal analysis showed that even though it moved across all five Brazilian regions, circulation of the B.1.1.28 variant was more frequently observed in the southeastern states [Figure 5A]. As expected, most viral exchange events occurred mainly between SJdRP and the Southeast and South regions; moreover, SJdRP exported the B.1.1.28 lineage essentially to southeastern cities [Figure 5B]. The B.1.1.33 lineage circulated similarly across the five Brazilian regions [Figure 5C]. We noted a predominance of B.1.1.33 importation and exportation events between SJdRP and the Southeast and Northeast regions [Figure 5D]. 

Compared to the two other lineages, VOI Zeta exhibited the most intense spread in the country. Despite its important role in the Brazilian epidemiological scenario from October 2020, our analysis showed that the lineage was moving across the country months earlier [Figure 5E]. We found VOI Zeta exportations from SJdRP to the five Brazilian regions, especially to the Northeast, although the majority of the lineage exchanges occurred between the Southeast and South regions [Figure 5F].

### 3.5. Defining Mutations Frequency 

To confirm the early circulation of the Zeta and N.9 variants in SJdRP in March and April 2020, respectively, we conducted an analysis to detect the presence of defining mutations in their genomes [Supplementary Tables S7 and S8]. Our findings revealed that the three defining mutations of the Zeta variant were present with a frequency of 100% [Table 1]. Similarly, among the six defining mutations of the N.9 variant, five exhibited a frequency of 100%, while one mutation located in ORF7b was identified with a frequency of 79% [Table 2].

## 4. Discussion

After the first introduction of SARS-CoV-2 in Brazil, subsequent viral importations from different countries occurred, leading to the cocirculation of multiple SARS-CoV-2 lineages across Brazil throughout 2020 [6]. We have corroborated these findings with our detection of 10 different lineages that were circulating in SJdRP from March to November 2020. As shown previously, the diversity of lineages circulating in the city decreased in 2021 due to the dominance of VOCs associated with the implementation of the vaccination policy [24].

Before the emergence of VOIs and VOCs, B.1.1.28 and B.1.1.33 were the most prevalent variants circulating in Brazil [4]. Similarly, our results showed that together, B.1.1.33 and B.1.1.28 lineages represented more than 87% of the genomes identified in SJdRP. Previous studies showed that the B.1.1.28 and B.1.1.33 lineages circulated primarily in the Southeast region of Brazil [4,25]. Reinforcing these findings, our spatiotemporal analysis showed that the Southeast region was the main contributor to the spread of B.1.1.28 and B.1.1.33 across the country, and that SJdRP acted as an important source of viral exportation for these variants to the five Brazilian regions. 

We also detected the N.9 variant in SJdRP. Our analysis revealed that the defining mutations of the variant were identified with an occurrence frequency of 79% across the study sequences, which happened due to the low depth of some genomes in specific regions. N.9 diverged from the B.1.1.33 variant in April 2020, seven months before it was first detected in São Paulo state and three months before the time that had been estimated as its origin [26]. These results are supported by previous studies that suggest viruses were locally circulating before the detection of disease cases [27,28,29]. These findings also emphasize how the absence of constant genomic surveillance makes it difficult to detect new viral introductions and determine their sources early.

Studies have shown that Zeta diverged from the B.1.1.28 lineage [7]. Additionally, the presence of mutations along the genome that caused an increase in viral fitness led to Zeta’s classification as a VOI [30,31,32]. The three defining mutations in ORF1ab, spike and nucleotide genes of the variant were identified with an occurrence frequency of 100% across the sequences generated in the present study. Previous studies showed that the variant emerged in the Brazilian COVID-19 pandemic scenario in 2020, before the emergence of VOC Gamma [7,8]. Our phylogeography analysis indicates that Zeta migration occurred from the Southeast and South regions to the rest of Brazil, as demonstrated previously [6]. Furthermore, we showed that SJdRP exported the variant to the five Brazilian regions, contributing to its spread over the country. 

Interestingly, the Zeta variant was first detected in Rio de Janeiro state in October 2020, although it is estimated to have originated in July 2020 [30]. Our findings indicate that, while the Zeta variant was first identified in SjdRP in March 2020, its emergence likely occurred earlier in Rio de Janeiro state. These results suggest a sampling bias in the genomic surveillance of SARS-CoV-2 variants in early 2020, which makes it difficult to make robust inferences about the divergence time of VOI Zeta. Additionally, a limitation of the present study is the lack of a Bayesian approach, which would provide a more accurate estimation of the divergence time of the Zeta variant in Brazil.

Banho et al. (2022) [24] showed that Zeta circulation became prevalent in SJdRP and was responsible for a peak of infections from November 2020, which indicates that VOI Zeta circulated at a low frequency for several months but accelerated at a certain point, impacting the frequency and the number of COVID-19 cases observed. These results reinforce the need for and importance of continuous genomic monitoring of SARS-CoV-2 lineages to better understand the virus evolution and spread, as well as the potential for existing variants to become dominant and impact public health.

## 5. Conclusions

We described the circulation dynamics of SARS-CoV-2 variants from March to November 2020 in a medium-sized city in São Paulo state that played an important role within the context of the COVID-19 pandemic in Brazil. We identified a variety of lineages that cocirculated in the municipality during 2020. Phylogenetic reconstructions suggested that the Zeta and N.9 variants circulated in Brazil earlier than previously reported. Phylogeography analyses revealed the SJdRP contribution to the spread of SARS-CoV-2 across the country. Moreover, implementing local genomic surveillance is critical to understanding epidemic patterns and helping governments make swifter decisions during public health emergencies.

## Figures and Tables

**Table 1:** Frequency of occurrence of the Zeta variant defining mutations in sequences generated in the present study.

Gene	Nucleotide	Amino Acid	Frequency (%)
ORF1ab	T10667G	L3468V	100
Spike	G23012A	E484K	100
N	G28628T	A119S	100

**Table 2:** Frequency of occurrence of the N.9 variant defining mutations in sequences generated in the present study.

Genome Position	Nucleotide	Amino Acid	Frequency (%)
ORF1a	G1264T	-	100
ORF1a	C7600T	-	100
ORF1a	C7851T	A2529V	100
ORF1a	T11078C	F3605L	100
Spike	G23012A	E484K	100
ORF7b	A27853C	E33A	79

## Data Availability

We declare that the main data supporting our findings are available within the article and its Supplementary Files. Code for genome assembly is publicly available in https://github.com/nf-core, accessed on 24 February 2022. Extra data are available from the corresponding author upon request.

## Supplementary Materials

The following supporting information can be downloaded at: https://www.mdpi.com/article/10.3390/pathogens13121069/s1, Figure S1: Number of new COVID-19 cases and deaths in SJdRP, February–December 2020; Figure S2: Average isolation rate in SJdRP from February to December 2020; Figure S3: Sociodemographic data for the sample population; Figure S4: Geographic distribution of B.1.1.28 lineage in SJdRP, March–November 2020; Figure S5: Geographic distribution of B.1.1.33 lineage in SJdRP, March–November 2020; Figure S6: Geographic distribution of VOI Zeta in SJdRP, March–November 2020; Figure S7: SARS-CoV-2 Maximum Likelihood (ML) analysis of VOI Zeta; Figure S8: Root-to-tip regression analysis of Zeta variant using TempEst (v.1.5.3); Figure S9: SARS-CoV-2 Maximum Likelihood (ML) analysis of N.9 variant; Figure S10: Root-to-tip regression analysis of N.9 variant using TempEst (v.1.5.3); Table S1: SARS-CoV-2 whole genome sequences generated in the present study and retrieved from the GISAID database (https://gisaid.org) used for Maximum Likelihood (ML) analysis based on complete genome sequences from SJdRP; Table S2: SARS-CoV-2 whole genome sequences generated in the present study and retrieved from the GISAID database (https://gisaid.org) used for Maximum likelihood (ML) analysis based on complete genome sequences of VOI Zeta; Table S3: SARS-CoV-2 whole genome sequences generated in the present study and retrieved from the GISAID database (https://gisaid.org) used for phylogenetic and phylogeographic analyses of the B.1.1.28 lineage; Table S4: SARS-CoV-2 whole genome sequences generated in the present study and retrieved from the GISAID database (https://gisaid.org) used for phylogenetic and phylogeography analyses of the B.1.1.33 lineage; Table S5: SARS-CoV-2 whole genome sequences generated in the present study and retrieved from the GISAID database (https://gisaid.org) used for phylogeography analyses of VOI Zeta; Table S6: SARS-CoV-2 whole genome sequences generated in the present study and retrieved from the GISAID database (https://gisaid.org) used for Maximum likelihood (ML) analysis based on complete genome sequences of N.9 variant; Table S7: SARS-CoV-2 whole genome sequences generated in the present study classified as Zeta variant and retrieved from the GISAID database (https://gisaid.org) used for mutation analysis; Table S8: SARS-CoV-2 whole genome sequences generated in the present study classified as N.9 variant and retrieved from the GISAID database (https://gisaid.org) used for mutation analysis; Table S9: SARS-CoV-2 whole genome sequences generated in the present study.
