# Supplementary material for: Spatiotemporal Dynamics of SARS-CoV-2 Variants During the First Year of the Pandemic Highlight the Earlier Emergence of the Zeta Variant of Interest in Brazil
Source: Pathogens. 2024 Dec 6;13(12):1069. doi: 10.3390/pathogens13121069 (PMC11728640; doi:10.3390/pathogens13121069)
Supplement: Supplementary file 1 [file Supplementary_Figures_final_version.pdf › Supplementary_Figures_final_version.pdf]

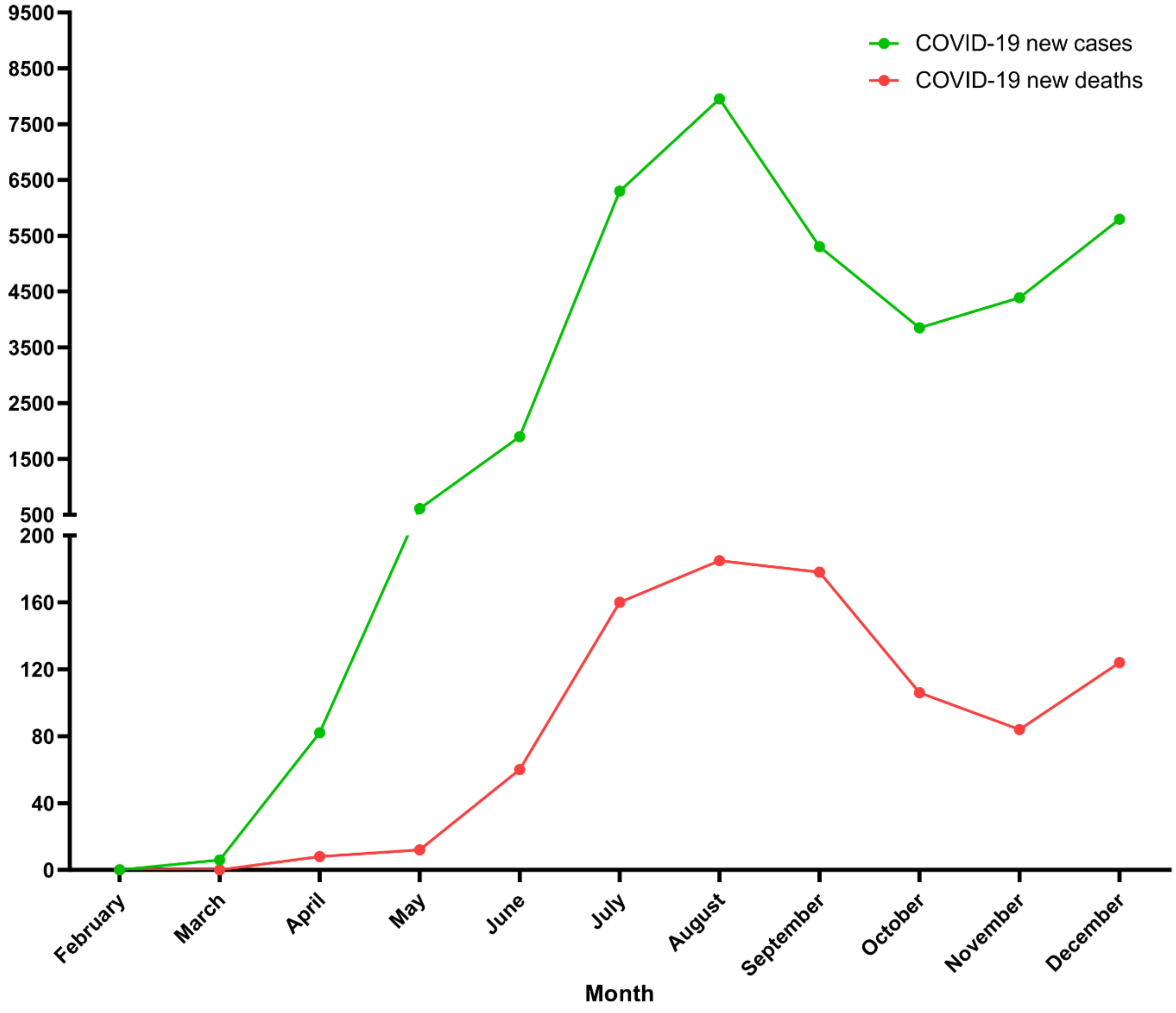

Figure S1. Number of new COVID-19 cases and deaths in SJdRP, February–December 2020. Data source: https://infoms.saude.gov.br/extensions/covid-19_html/covid-19_html.html.

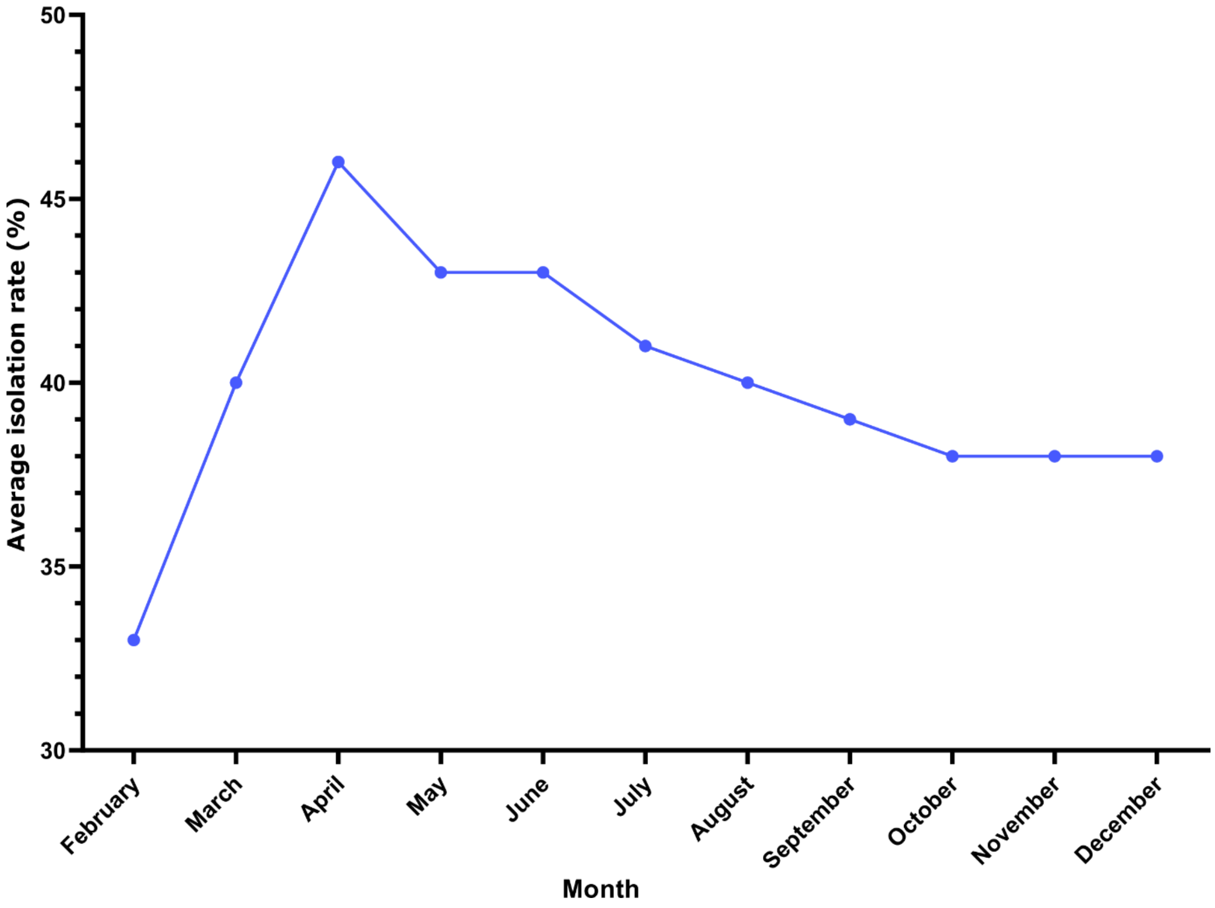

Figure S2. Average isolation rate in SJdRP from February to December 2020. Data source: https://infoms.saude.gov.br/extensions/covid-19_html/covid-19_html.html.

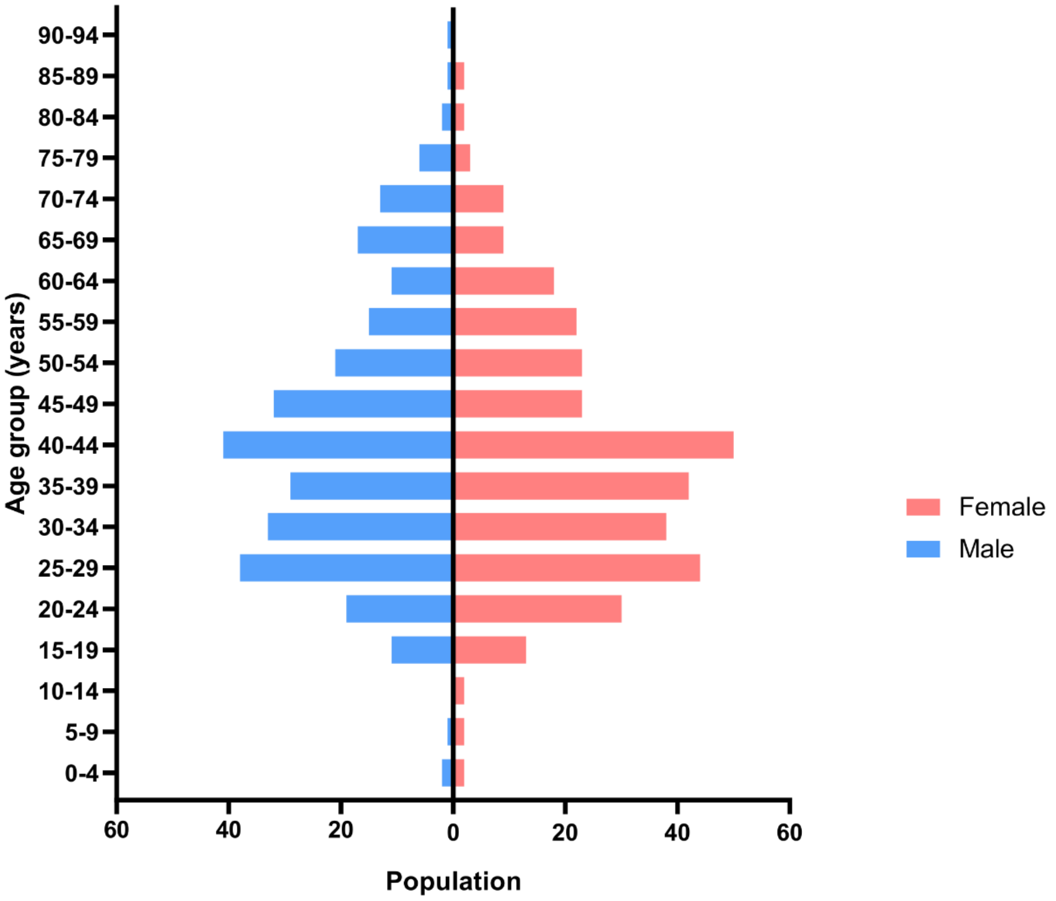

Figure S3. Sociodemographic data for the sample population. Sociodemographic data from the population providing the nasopharyngeal samples used in the present study.

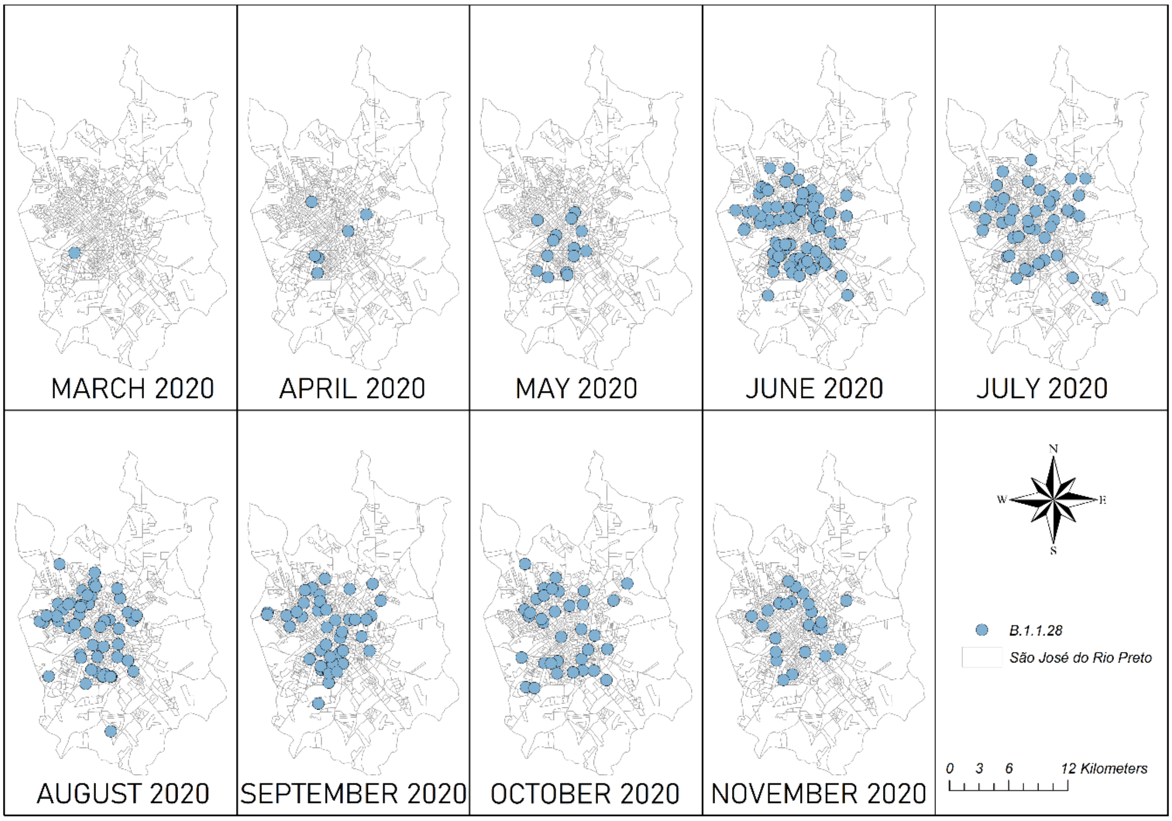

Figure S4. Geographic distribution of B.1.1.28 lineage in SJdRP, March–November 2020. Sampling location and collection dates were used to create the maps in QGIS v.3.36.0-1 software (http://qgis.org).

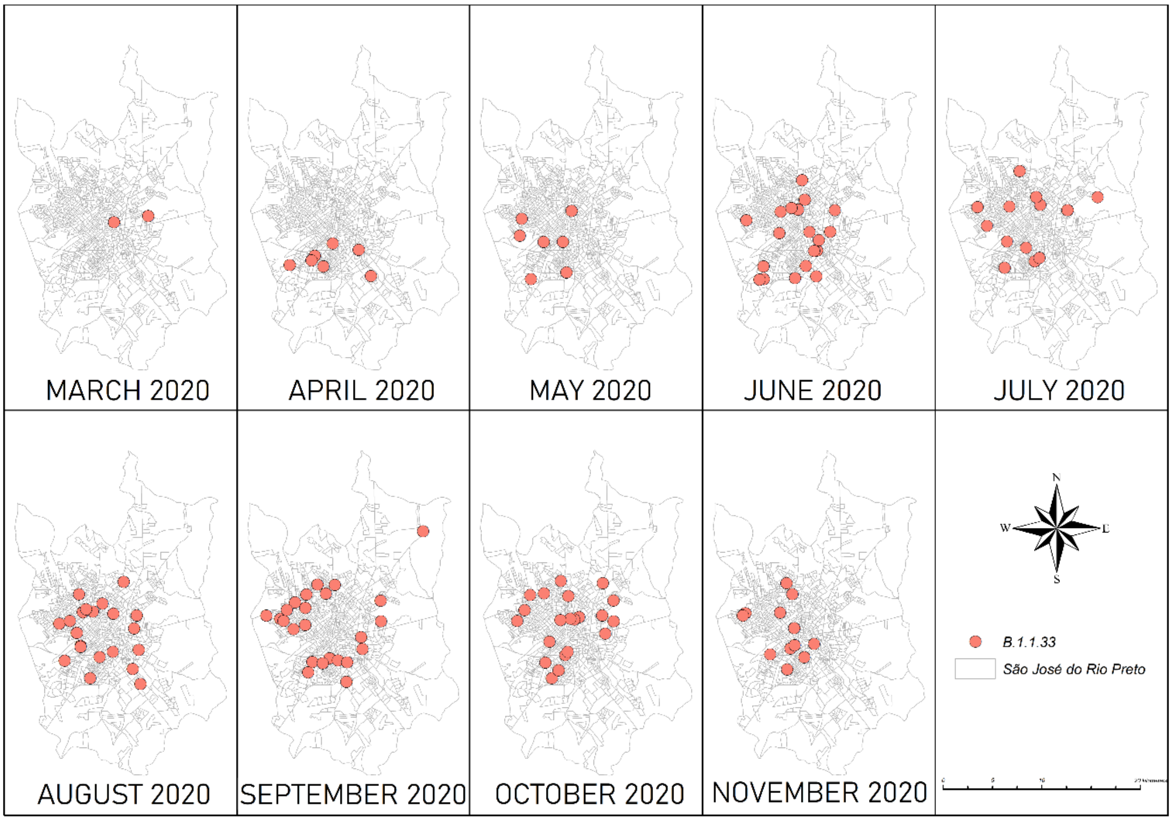

Figure S5. Geographic distribution of B.1.1.33 lineage in SJdRP, March–November 2020. Sampling location and collection dates were used to create the maps in QGIS v.3.36.0-1 software (http://qgis.org).

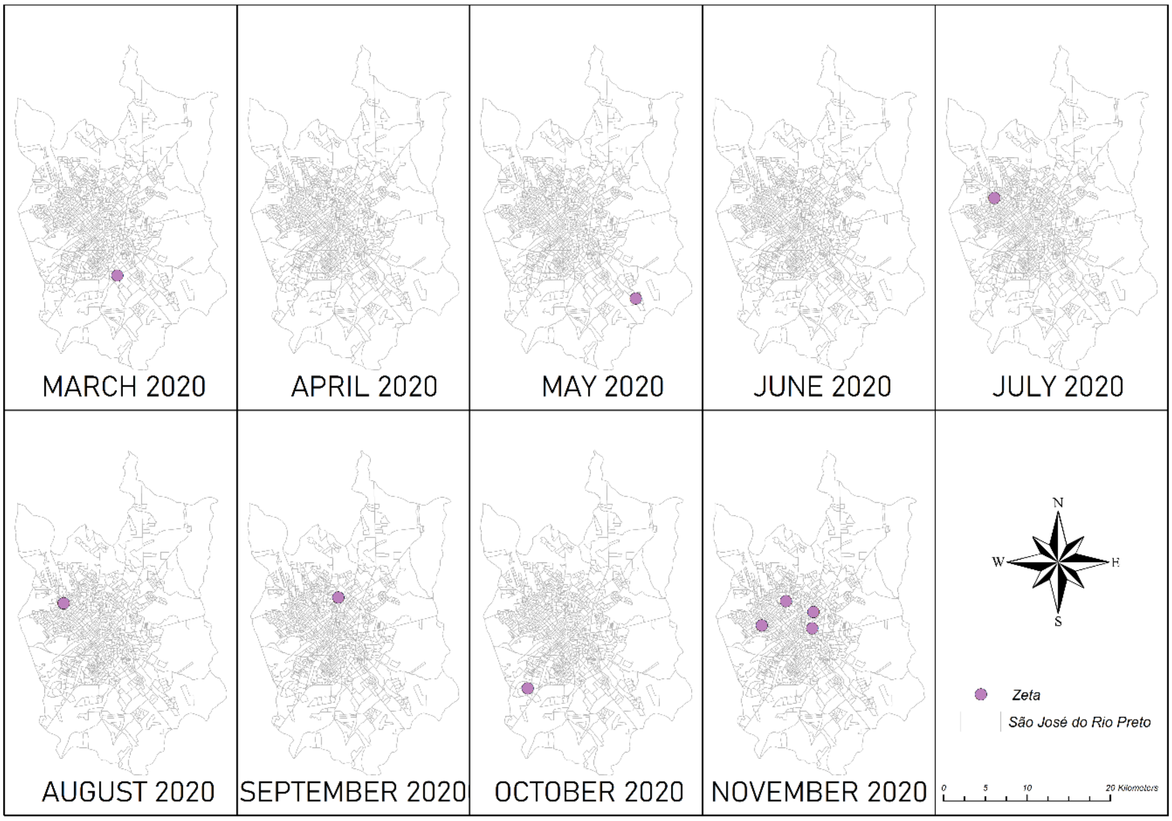

Figure S6. Geographic distribution of VOI Zeta in SJdRP, March–November 2020. Sampling location and collection dates were used to create the maps in QGIS v.3.36.0-1 software (http://qgis.org).

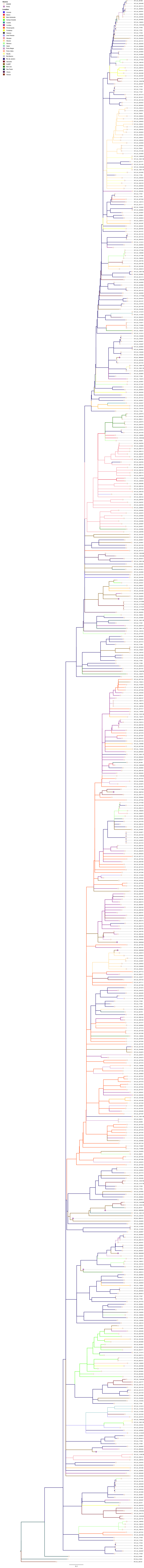

Source
GISAID
Study
Location
Aracaju
Belem
Belo Horizonte
Campo Grande
Cuiaba
Curitiba
Florianopolis
Fortaleza
Goiania
Joao Pessoa
Macapa
Maceio
Manaus
Natal
Porto Alegre
Porto Velho
Recife
Rio Branco
Rio de Janeiro
Salvador
SJdRP
Sao Luis
Sao Paulo
Vitoria
Wuhan

Figure S7. SARS-CoV-2 Maximum Likelihood (ML) analysis of VOI Zeta. TreeTime mugration v.0.9.3 was used to map the locations to tips and internal nodes from the ML tree topology. Tips are colored according to sequences source, and nodes are colored according to sequences location.

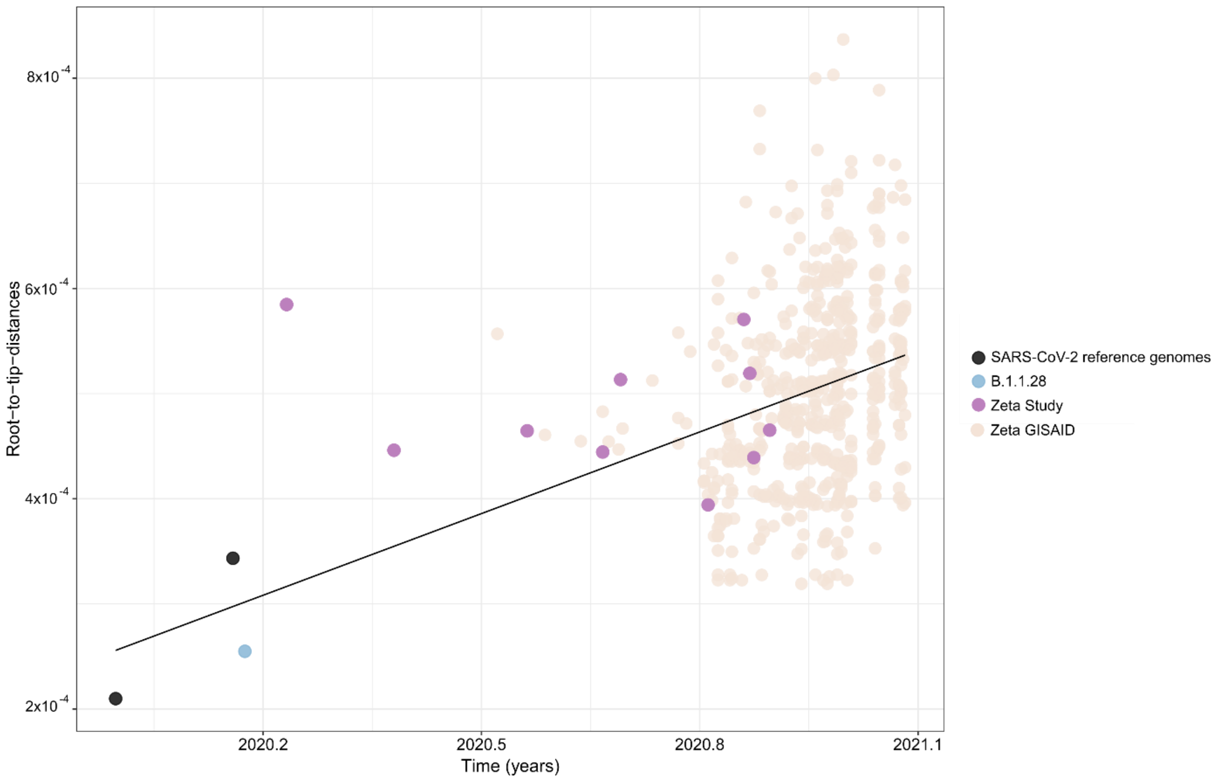

Figure S8. Root-to-tip regression analysis of Zeta variant using TempEst (v.1.5.3). The plot represents linear regression of root-to-tip divergence against sampling time for the Zeta variant dataset [Table S2]. Colors indicate SARS-CoV-2 reference genomes, the first genome of B.1.1.28 lineage detected in Brazil, VOI Zeta genomes generated in this study and VOI Zeta genomes retrieved from the GISAID database (https://gisaid.org).

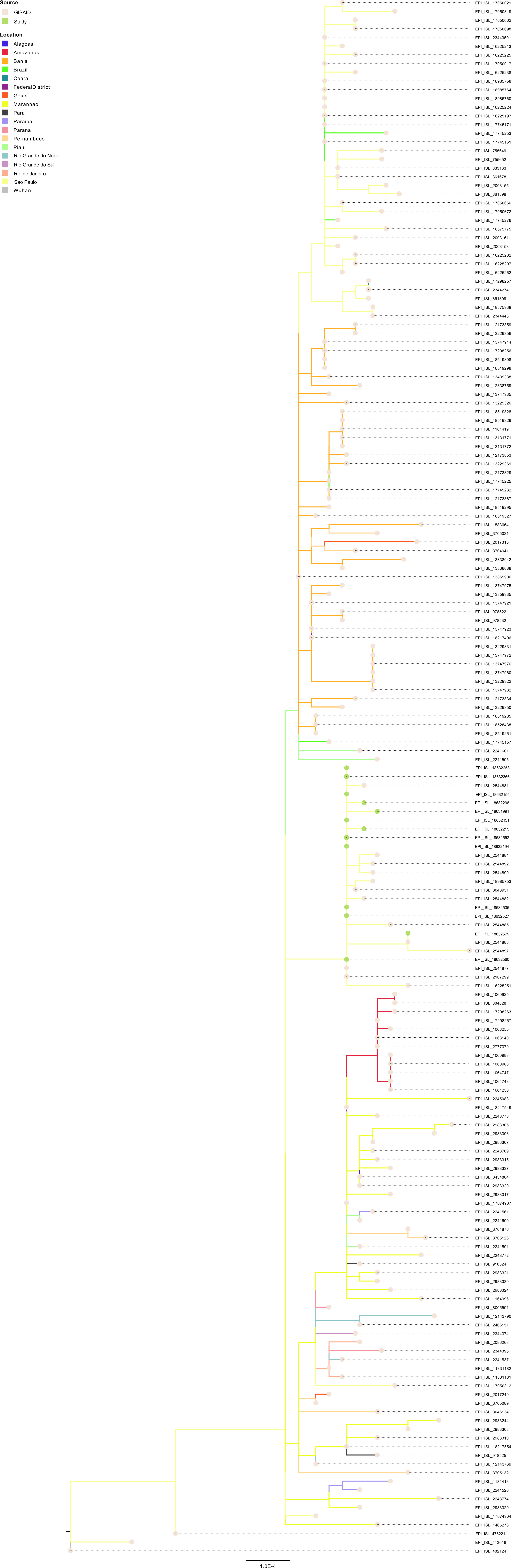

Source
GISAID
Study
Location
Alagoas
Amazonas
Bahia
Brazil
Ceara
FederalDistrict
Goias
Maranhao
Para
Paraiba
Parana
Pernambuco
Piaui
Rio Grande do Norte
Rio Grande do Sul
Rio de Janeiro
Sao Paulo
Wuhan
EPI_ISL_17050319
EPI_ISL_17050662
EPI_ISL_17050698
EPI_ISL_2344359
EPI_ISL_16225213
EPI_ISL_16225225
EPI_ISL_17050017
EPI_ISL_16225238
EPI_ISL_18985758
EPI_ISL_18985764
EPI_ISL_18985760
EPI_ISL_16225224
EPI_ISL_16225197
EPI_ISL_17745171
EPI_ISL_17745253
EPI_ISL_17745161
EPI_ISL_755649
EPI_ISL_755652
EPI_ISL_833163
EPI_ISL_861678
EPI_ISL_2003155
EPI_ISL_861898
EPI_ISL_17050666
EPI_ISL_17050672
EPI_ISL_17745276
EPI_ISL_18575775
EPI_ISL_2003161
EPI_ISL_2003153
EPI_ISL_16225202
EPI_ISL_16225207
EPI_ISL_16225262
EPI_ISL_17298257
EPI_ISL_2344274
EPI_ISL_861899
EPI_ISL_18875938
EPI_ISL_2344443
EPI_ISL_12173859
EPI_ISL_13229356
EPI_ISL_13747914
EPI_ISL_17298256
EPI_ISL_18519308
EPI_ISL_18519298
EPI_ISL_13439338
EPI_ISL_12838759
EPI_ISL_13747935
EPI_ISL_13229326
EPI_ISL_18519328
EPI_ISL_18519329
EPI_ISL_1181419
EPI_ISL_13131771
EPI_ISL_13131772
EPI_ISL_12173853
EPI_ISL_13229361
EPI_ISL_12173829
EPI_ISL_17745225
EPI_ISL_17745232
EPI_ISL_12173867
EPI_ISL_18519295
EPI_ISL_18519327
EPI_ISL_1583664
EPI_ISL_3705021
EPI_ISL_2017315
EPI_ISL_3704941
EPI_ISL_13838042
EPI_ISL_13838088
EPI_ISL_13859906
EPI_ISL_13747975
EPI_ISL_13859935
EPI_ISL_13747921
EPI_ISL_978522
EPI_ISL_978532
EPI_ISL_13747923
EPI_ISL_18217496
EPI_ISL_13229331
EPI_ISL_13747972
EPI_ISL_13747976
EPI_ISL_13747960
EPI_ISL_13229322
EPI_ISL_13747982
EPI_ISL_12173834
EPI_ISL_13229350
EPI_ISL_18519285
EPI_ISL_18528438
EPI_ISL_18519261
EPI_ISL_17745157
EPI_ISL_2241601
EPI_ISL_2241595
EPI_ISL_18632253
EPI_ISL_18632366
EPI_ISL_2544881
EPI_ISL_18632155
EPI_ISL_18632298
EPI_ISL_18631991
EPI_ISL_18632451
EPI_ISL_18632215
EPI_ISL_18632552
EPI_ISL_18632194
EPI_ISL_2544884
EPI_ISL_2544892
EPI_ISL_2544890
EPI_ISL_18985753
EPI_ISL_3048951
EPI_ISL_2544882
EPI_ISL_18632535
EPI_ISL_18632527
EPI_ISL_2544885
EPI_ISL_18632579
EPI_ISL_2544888
EPI_ISL_2544897
EPI_ISL_18632560
EPI_ISL_2544877
EPI_ISL_2107299
EPI_ISL_16225251
EPI_ISL_1060925
EPI_ISL_804828
EPI_ISL_17298263
EPI_ISL_17298267
EPI_ISL_1068255
EPI_ISL_1068140
EPI_ISL_2777370
EPI_ISL_1060983
EPI_ISL_1060988
EPI_ISL_1064747
EPI_ISL_1064743
EPI_ISL_1661250
EPI_ISL_2245083
EPI_ISL_18217549
EPI_ISL_2248773
EPI_ISL_2983305
EPI_ISL_2983306
EPI_ISL_2983307
EPI_ISL_2248769
EPI_ISL_2983315
EPI_ISL_2983337
EPI_ISL_3434804
EPI_ISL_2983320
EPI_ISL_2983317
EPI_ISL_17074907
EPI_ISL_2241561
EPI_ISL_2241600
EPI_ISL_3704876
EPI_ISL_3705126
EPI_ISL_2241591
EPI_ISL_2248772
EPI_ISL_918524
EPI_ISL_2983321
EPI_ISL_2983330
EPI_ISL_2983324
EPI_ISL_1164996
EPI_ISL_8005591
EPI_ISL_12143790
EPI_ISL_2466151
EPI_ISL_2344374
EPI_ISL_2086268
EPI_ISL_2344395
EPI_ISL_2241537
EPI_ISL_11331182
EPI_ISL_11331181
EPI_ISL_17050312
EPI_ISL_2017249
EPI_ISL_3705089
EPI_ISL_3046134
EPI_ISL_2983244
EPI_ISL_2983308
EPI_ISL_2983310
EPI_ISL_18217554
EPI_ISL_918525
EPI_ISL_12143769
EPI_ISL_3705132
EPI_ISL_1181416
EPI_ISL_2241526
EPI_ISL_2248774
EPI_ISL_2983329
EPI_ISL_17074904
EPI_ISL_1465278
EPI_ISL_476221
EPI_ISL_413016
EPI_ISL_402124
1.0E-4

Figure S9. SARS-CoV-2 Maximum Likelihood (ML) analysis of N.9 variant. The phylogenetic analysis was conducted in IQ-TREE v2.2.0. The phylogenetic tree was reconstructed using GTR+F+I as the best nucleotide substitution model. The reliability of branching patterns was tested by combining Ultrafast Bootstrap (UFBoot) and SH-like Approximate Likelihood-ratio (SH-aLRT) tests, with 10,000 replicates each. The dataset used comprises a total of 179 genomes. TreeTime mugration v.0.9.3 was used to map the locations to tips and internal nodes from the ML tree topology. Tips are colored according to sequences source, and nodes are colored according to sequences location.

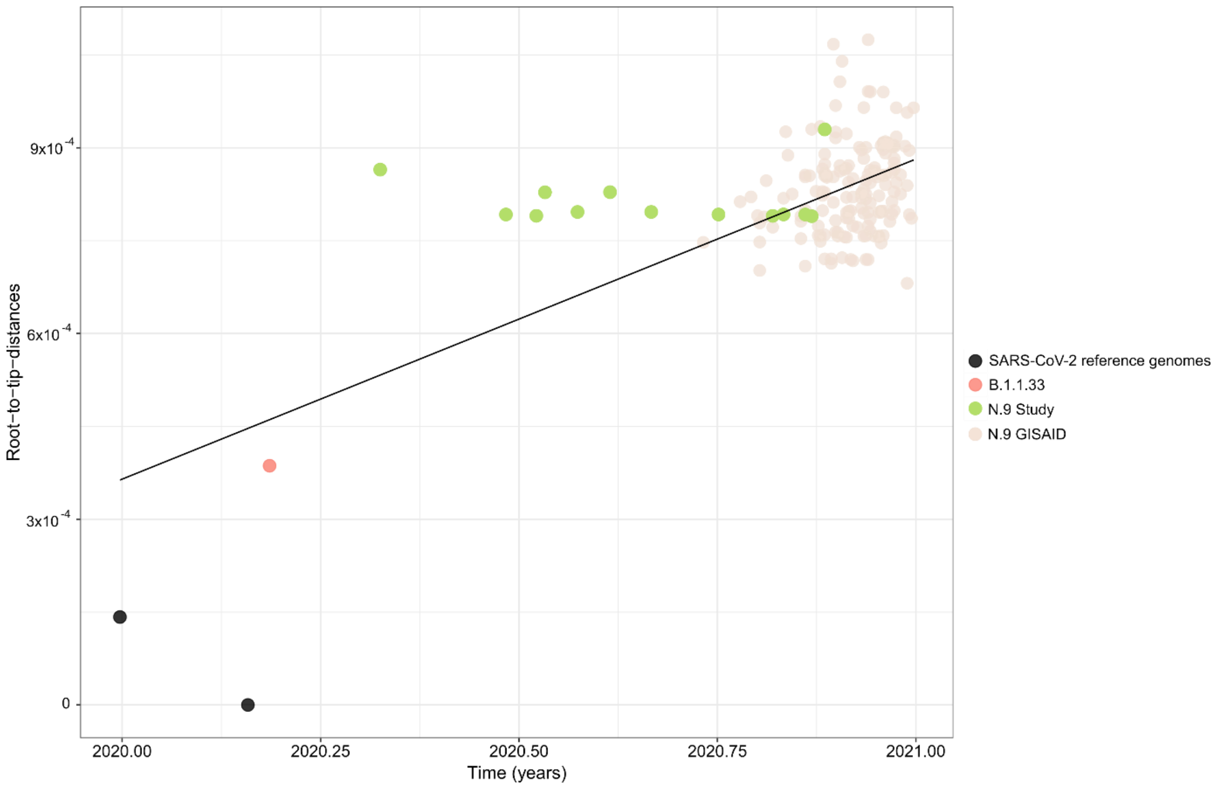

Figure S10. Root-to-tip regression analysis of N.9 variant using TempEst (v.1.5.3). The plot represents linear regression of root-to-tip divergence against sampling time for the N.9 variant dataset [Table S9]. Colors indicate SARS-CoV-2 reference genomes, the first genome of B.1.1.33 lineage detected in Brazil, N.9 variant genomes generated in this study and N.9 variant genomes retrieved from the GISAID database (https://gisaid.org).
